# Effects of Lysophospholipids on the Antioxidant Capacity, Digestive Performance, and Intestinal Microbiota of *Litopenaeus vannamei*

**DOI:** 10.3390/biology14010090

**Published:** 2025-01-17

**Authors:** Hailiang Yan, Yun Wang, Hong Liang, Yafei Duan, Jun Wang, Chuanpeng Zhou, Zhong Huang

**Affiliations:** 1 College of Fisheries and Life Science, Shanghai Ocean University, Shanghai 201306, China; hailiangyan813@163.com (H.Y.); lianghong99001122@163.com (H.L.); 2 Key Laboratory of Aquatic Product Processing, Ministry of Agriculture and Rural Affairs, South China Sea Fisheries Research Institute, Chinese Academy of Fishery Sciences, Guangzhou 510300, China; duanyafei89@163.com (Y.D.); junnywang@163.com (J.W.); chpzhou@163.com (C.Z.); 3 Key Laboratory of Efficient Utilization and Processing of Marine Fishery Resources of Hainan Province, Hainan Engineering Research Center of Deep-Sea Aquaculture and Processing, Sanya Tropical Fisheries Research Institute, Sanya 572018, China; 4 Guangdong Provincial Key Laboratory of Fishery Ecology and Environment, South China Sea Fisheries Research Institute, Chinese Academy of Fisheries Sciences, Guangzhou 510300, China; 5 Shenzhen Base of South China Sea Fisheries Research Institute, Chinese Academy of Fishery Sciences, Shenzhen 518121, China; huangzhongnhs@163.com

**Keywords:** *Litopenaeus vannamei*, lysophospholipids, antioxidant capacity, digestion, intestinal microbiota

## Abstract

This study seeks to assess the impact of varying concentrations of lysophospholipids on the antioxidant capacity, digestive performance, and intestinal microbiota of *L. vannamei*. A total of 840 shrimp, with an average initial weight of 2.22 ± 0.11 g, were randomly distributed across 28 aquaculture tanks (30 shrimp per tank), organized into 7 distinct treatment groups, each comprising 4 replicates. The control group (DL2) was administered a basal diet formula supplemented with 2% soy lecithin, whereas the experimental groups received lysophospholipids at varying concentrations of 0% (RL0), 0.1% (RL0.1), 0.5% (RL0.5), 1% (RL1), 1.5% (RL1.5), and 2% (RL2). The results revealed that the total antioxidant capacity (T-AOC) level in the RL0.1 group was significantly elevated compared to the DL2 and RL2 groups (*p* < 0.05). Furthermore, glutathione peroxidase (GSH-Px) and catalase (CAT) activities were markedly higher in the RL1 and RL1.5 groups relative to other groups (*p* < 0.05). Superoxide dismutase (SOD) activity exhibited a significant increase across all lysophospholipid-supplemented groups when compared to the DL2 group (*p* < 0.05). Malondialdehyde (MDA) levels were notably elevated in the RL0.5, RL1.5, and RL2 groups relative to the other groups (*p* < 0.05). Regarding antioxidant-related genes in the hemocytes, the relative expression levels of *Nrf1*, *Nrf2*, *GPx*, *SOD*, *CAT*, and *Hippo* were significantly higher in the RL0.5 and RL1 groups compared to the DL2 group (*p* < 0.05). Additionally, the relative expression levels of *GPx* and *SOD* were significantly elevated in the RL2 group compared to the DL2 group (*p* < 0.05). In the intestinal tract, the activities of trypsin and α-amylase (AMS) were significantly elevated in the RL0.1, RL0.5, and RL1 groups compared to the DL2 group (*p* < 0.05). Additionally, the RL0.1 group demonstrated significantly higher lipase (LPS) activity than the other groups supplemented with lysophospholipids (*p* < 0.05). Furthermore, the relative expression levels of the *trypsin* and *α-amylase* genes were significantly increased in the RL1 and RL1.5 groups in comparison to the DL2 group (*p* < 0.05). Sequencing analysis of the intestinal microbiota indicated that the incorporation of lysophospholipids resulted in an enhancement of the composition and structure of the intestinal microbiota. The functional abundance of the intestinal microbiome was primarily enriched in metabolic pathways. Overall, the incorporation of lysophospholipids into the diet exerted a beneficial effect on the antioxidant capacity, digestive performance, and intestinal microbiota of *L. vannamei*, especially with the supplementation of 0.1% lysophospholipids.

## Simple Summary

In comparison to natural phospholipids, lysophospholipids demonstrate increased hydrophilicity and enhanced emulsifying properties. Nonetheless, there is a paucity of information regarding the impact of lysophospholipid supplementation on *Litopenaeus vannamei* within crustacean research. This study examines the effects of dietary lysophospholipid supplementation on the antioxidant capacity, digestive performance, and intestinal microbiota of *L. vannamei*. The findings suggest that dietary supplementation with lysophospholipids confers beneficial effects on *L. vannamei*. These findings offer a theoretical foundation for the effective use of lysophospholipids in *L. vannamei* aquaculture and establish a basis for future research on its metabolic mechanisms.

## 1. Introduction

China stands as the preeminent global producer of aquatic products, achieving an aquaculture fish production of 49.9 million tons in 2020, which constitutes 57.03% of the worldwide total [1]. Aquatic animals are a critical source of protein and micronutrients in China, significantly contributing to food security and nutritional supply. Since the 1990s, *Litopenaeus vannamei*, a shrimp species belonging to the Penaeidae family, has been extensively cultivated in various regions across the country [2]. It possesses several advantages, including robust adaptability, extensive salinity tolerance, rapid growth, strong disease resistance, palatable taste, and high meat yield upon processing [3]. As reported in the 2022 China Fisheries Yearbook, *L. vannamei* production reached 1.978 million tons, accounting for 86.9% of the total domestic shrimp production [4].

The ongoing expansion of aquaculture, coupled with the widespread adoption of artificial feed, has led to a heightened demand for feed protein. Nonetheless, the availability of high-quality feed protein resources is diminishing, thereby presenting substantial challenges to the pricing and valuation of aquaculture feeds amidst significant cost pressures. Fat, as a high-energy source, can diminish the reliance on protein for energy, thereby permitting a greater allocation of protein toward tissue synthesis and growth [5]. Consequently, due to the protein-sparing effect of fat, it has been observed that appropriately increasing the fat content in feed can effectively conserve protein and enhance the growth and health of fish [6]. However, high-fat diets may result in excessive fat accumulation in aquatic animals, leading to conditions such as fatty liver and lipid metabolism disorders [7], which can subsequently impair growth and overall health.

Therefore, the strategic augmentation of fat content in feed to enhance protein utilization holds sustainable importance for advancing the sustainable development of aquaculture. Soybean phospholipids are natural phospholipids isolated from the extraction process of soybean oil. They are produced in large quantities, from a wide range of sources, and at low prices, and are often used as nutritional supplements and emulsifiers in feed [8]. Lysophospholipids, produced through the hydrolysis of phospholipids by phospholipase [9], exhibit enhanced hydrophilicity and are incorporated into feed in smaller amounts compared to conventional phospholipids. Lysophospholipids, characterized as monoacyl phospholipids due to the absence of one fatty acid chain, exhibit a heightened hydrophilicity relative to conventional phospholipids. This increased hydrophilicity enhances their efficacy as emulsifiers, facilitating the formation of mixed micelles and thereby improving the absorption and utilization efficiency of fats [10]. The utilization of lysophospholipids in animal nutrition has been extensively investigated, with a particular focus on their potential to enhance growth performance [11,12]. Empirical evidence indicates that dietary supplementation with lysophospholipids can significantly improve nutrient digestibility and growth metrics in livestock species, including poultry and pigs [13]. Recent studies have highlighted the significant role of lysophospholipids in aquatic animals [14,15,16,17], demonstrating their efficacy in enhancing growth, promoting intestinal digestion, augmenting antioxidant capacity, and exerting lipid-lowering effects, which collectively contribute to improved health effects. Consequently, lysophospholipids present promising potential as feed additives in aquaculture.

This study investigates the effects of lysophospholipid supplementation in shrimp feed on the antioxidant capacity, digestive performance, and intestinal health of *L. vannamei*. The aim is to ascertain the optimal level of lysophospholipids inclusion in the diet and to elucidate the mechanisms through which lysophospholipids improve feed utilization in *L. vannamei*.

## 2. Materials and Methods

### 2.1. Ethical Statement

This study was conducted in compliance with the animal use protocol NHDF2023-12 and received approval from the Experimental Animal Welfare and Ethics Committee of the South China Sea Fisheries Research Institute, Chinese Academy of Fishery Sciences.

### 2.2. Experimental Materials

The formulation and nutritional composition of the experimental diets are detailed in Table 1. Seven experimental diets were developed using *L. vannamei* as the foundational base. The control group (DL2) received a supplementation of 2% soybean lecithin [18], whereas the experimental groups were administered varying concentrations of lysophospholipids: 0% (RL0 group), 0.1% (RL0.1 group), 0.5% (RL0.5 group), 1% (RL1 group), 1.5% (RL1.5 group), and 2% (RL2 group). Each group consisted of four replicates. The ingredients were finely ground into a powder, passed through an 80-mesh sieve, and meticulously combined with oil and water. The resulting feed mixture was extruded into elongated dough strips with diameters of 1.0 mm and 1.5 mm utilizing a twin-screw extruder (F-26, South China University of Technology, Guangzhou, China), followed by pelletization using a granulator (G-500, South China University of Technology, Guangzhou, China). The pellets underwent thermal treatment in an oven at 90 °C for 90 min, followed by air-drying to approximate moisture content of 10%. Subsequently, they were stored in a freezer at −20 °C.

### 2.3. Experimental Design and Management

The white shrimp utilized in this experiment were temporarily accommodated at the Shenzhen base of the South China Sea Fisheries Research Institute, Chinese Academy of Fishery Sciences. Healthy and uniform shrimp with an average body weight of 2.22 ± 0.11 g were selected. A total of 860 white shrimp were randomly distributed across 28 fiberglass tanks (500 L, with a bottom area of 0.5 m^2^). Each experimental group was allocated 4 tanks, with 30 shrimp per tank. Throughout the experimental period, the rearing water was maintained at a temperature of 29.8 ± 1.4 °C, with a salinity of 29.4 ± 0.8, a pH level of 8.0 ± 0.1, and dissolved oxygen concentrations exceeding 6.0 mg/L. The feeding regimen was established at 2% to 4% of the initial total body weight of the shrimp per tank, feeding thrice daily at 8:00, 17:00, and 22:00. The rearing trial lasted for 56 days, during which the survival rate of the shrimp was systematically recorded. A feeding platform was installed in each tank to monitor feed consumption, and residual feed was observed one hour post-feeding. Adjustments to the feeding quantity were made in response to weather conditions and observed feeding behavior.

### 2.4. Collection and Preservation of Samples

Following the 56 day cultivation period, the shrimp underwent a 24 h fasting period. Subsequently, ten healthy and lively shrimp were randomly selected from each tank. The seawater on their carapaces was carefully dried using gauze. The shrimp bodies were then swabbed with 75% ethanol. Hemolymph samples were collected using a 1 mL syringe and mixed in a 1:1 ratio with an ACD anticoagulant (Regen Biotechnology Co., Ltd., No. R10202, Beijing, China). The hemolymph was subjected to centrifugation at 4 °C and 4000 rpm for 10 min, after which the supernatant was preserved at −80 °C. The resulting pelleted hemocytes were utilized for the extraction of total RNA. Following hemocyte collection, the intestinal tissue of the shrimp was immediately flash-frozen in liquid nitrogen and subsequently stored at −80 °C. For the analysis of intestinal microbiota, the intestines from four shrimp per tank were similarly flash-frozen in liquid nitrogen and stored at −80 °C.

### 2.5. Assessment of Antioxidant Enzymes Activity

The antioxidant enzymes activities of the hemolymph in white shrimp, including total antioxidant capacity (T-AOC), superoxide dismutase (SOD), catalase (CAT), glutathione peroxidase (GPx), malondialdehyde (MDA), and total protein (TP), were measured using commercial assay kits (Nanjing Jiancheng Bioengineering Institute, Nanjing, China).

### 2.6. Assessment of Intestinal Enzymes Activity

Intestinal tissue was homogenized in sterile phosphate-buffer saline (PBS) solution (0.86%, pH 7.4, 1:9, *w*/*v*) using a QIAGEN TissueLyser II homogenizer (Dusseldorf, Germany). This was followed by centrifugation at 3500 rpm for 15 min at 4 °C. The resultant supernatant was collected for subsequent enzyme activity assays [19]. The enzymatic activities of Trypsin, AMS, and LPS were quantified using commercially available assay kits from Nanjing Jiancheng Bioengineering Institute (Nanjing, China).

### 2.7. Real-Time PCR Analysis of Antioxidant and Digestive-Related Genes Expression Patterns

Samples preserved at −80 °C were subsequently thawed on ice, and RNA extraction was performed in accordance with the instructions provided by the Trizol reagent (Invitrogen, Shanghai, China). The synthesis of first-strand cDNA was conducted using the Evo M-MLV Reverse Transcription Premix Kit, following the manufacturer’s protocol (Accurate Biotechnology Code No. AG11728, Changsha, China).

Real-time PCR utilizing the SYBR Green method was conducted in accordance with the 2^−∆∆ct^ relative quantification technique [20], adhering to the protocol provided by the SYBR Green Pro Taq HS qPCR Premix Kit (Accurate Biotechnology Code No. AG11701, Changsha, China). The samples were prepared in PCR tubes, transferred to a 96-well PCR plate, briefly centrifuged, and then subjected to PCR amplification using a PCR detection system (Heal Force CG-02, Shanghai, China). The primers for real-time PCR were synthesized by Sangon Biotech Co., Ltd. (Shanghai, China), and the specific primers employed for the amplification are detailed in Table 2. The quantified result was analyzed by the following formulae:

∆ct = Target gene ct value − Reference gene ct value

∆∆ct value = ∆ct value of treatment group − ∆ct value of control group

### 2.8. Intestinal Microbial Community Analysis

Genomic DNA was isolated from intestinal microbiome samples using the OMEGA Soil DNA Kit (M5635-02) (Omega Bio-Tek, Norcross, GA, USA) and subsequently stored at −20 °C. Amplification of the bacterial 16S rRNA gene V3-V4 region was performed using the primers 338F (5′-ACTCCTACGGGAGGCAGCA-3′) and 806R (5′-GGACTACHVGGGTWTCTAAT-3′). The amplified products underwent purification and recovery through the application of magnetic beads, followed by fluorescence quantification utilizing the Quant-iT PicoGreen dsDNA Assay Kit on a Microplate Reader (BioTek, FLx800). Subsequent to sample quantification, sequencing libraries were construed employing Illumina’s TruSeq Nano DNA LT Library Prep Kit. Paired-end sequencing was executed on a NovaSeq sequencer using the NovaSeq 6000 SP Reagent Kit (San Diego, CA, USA). Alpha diversity for each sample was evaluated utilizing QIIME2 (2019.4), focusing on the distribution of Amplicon Sequence Variants (ASVs). Beta diversity variations and their statistical significance were determined through the application of distance matrices. Comparative analyses of species abundance compositions across groups were conducted, leading to the identification of marker species. Utilizing 16S rRNA sequencing data, pathway abundances were examined via the KEGG Pathway Database [http://www.genome.jp/kegg/pathway.html (accessed on 18 August 2024)] to discern differential pathways and predict the metabolic functions of the microbiota present in the samples.

### 2.9. Statistical Analysis

All data are presented as mean ± standard error and subjected to statistical analysis using SPSS 27.0. The assumptions of normality and homogeneity of variance were evaluated using the Kolmogorov–Smirnov and Levene’s tests, respectively. A one-way analysis of variance (ANOVA) was conducted, followed by Duncan’s multiple range test for post hoc comparisons. Statistical significance was determined at a threshold of *p* < 0.05. Karl– Pearson correlation analysis was performed using the BioDeep Platform [https://www.biodeep.cn (accessed on 6 November 2024)] to establish the relationship between different antioxidant and digestive indicators.

## 3. Results

### 3.1. Activity of Antioxidant Enzymes in Hemolymph

The antioxidant enzyme activity in the hemolymph of *L. vannamei* is shown in Table 3. As the lysophospholipid concentration increased from 0.1% to 2%, the activities of T-AOC and CAT initially increased, followed by a decline. In contrast, the activity of SOD exhibited a pattern similar to that of MDA content, characterized by an initial increase, a subsequent decrease, and a final increase. In the RL0.1 group, the T-AOC level was significantly elevated compared to the DL2 and RL2 groups (*p* < 0.05), while no significant differences were observed among the remaining groups (*p* > 0.05). The activities of GSH-Px and CAT were significantly higher in the RL1 and RL1.5 groups compared to other groups (*p* < 0.05). Additionally, SOD activities were significantly increased in all lysophospholipid-supplemented groups relative to the DL2 group (*p* < 0.05). The MDA content was significantly elevated in the RL0.5, RL1.5, and RL2 groups relative to all other groups (*p* < 0.05). Furthermore, the incorporation of 0.1% lysophospholipids markedly enhanced the hemolymph T-AOC capacity in shrimp. In contrast, supplementation with 0.5% to 1.5% lysophospholipids led to a significant increase in GSH-Px and CAT activities, as well as MDA content. The supplementation of 1.5% to 2% lysophospholipids resulted in a significant enhancement of SOD activity and MDA content. This suggests that lysophospholipids facilitate the activity of hemolymph antioxidant enzymes, with elevated concentrations notably increasing MDA levels.

### 3.2. Relative Expression Levels of Antioxidant Genes in the Hemocytes

**Figure 1:**
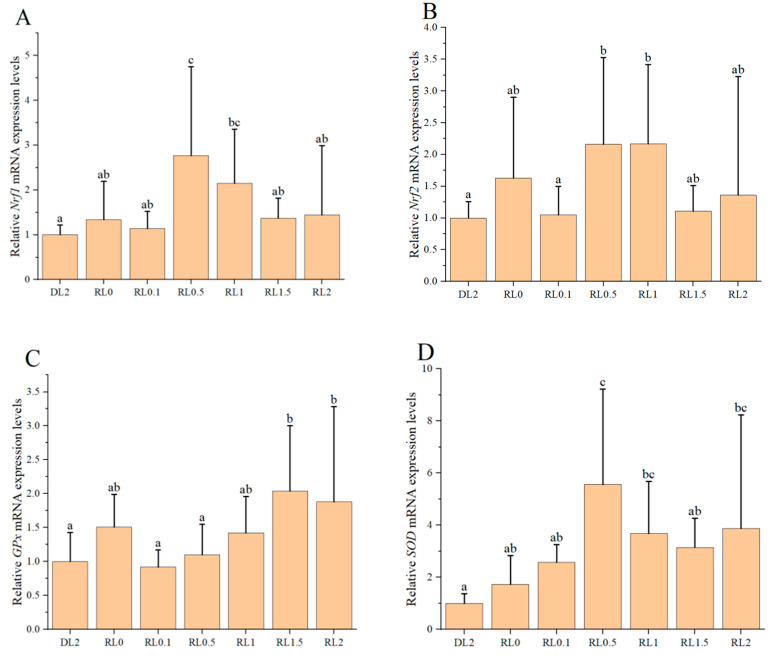

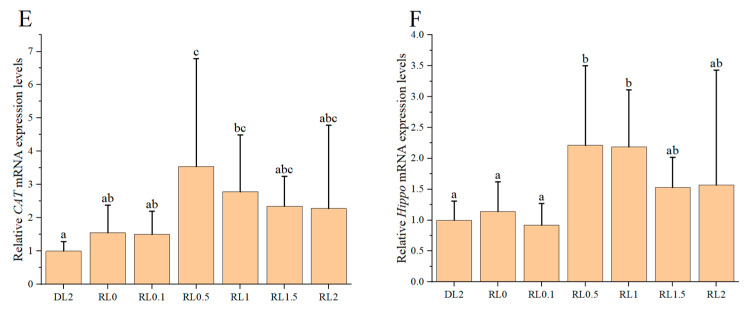
The impact of lysophospholipids on the relative expression levels of antioxidant genes in the hemocytes of shrimp. (**A**) *Nrf1*; (**B**) *Nrf2*; (**C**) *GPx*; (**D**) *SOD*; (**E**) *CAT*; (**F**) Hippo. The reference gene was *β-actin*. Different lowercase letters above the bars indicate statistically significant differences (*n* = 4, *p* < 0.05).

The relative expression levels of antioxidant genes in the hemocytes of shrimp are shown in Figure 1. Upon the addition of lysophospholipids ranging from 0.1% to 2%, there is an initial increase followed by a subsequent decrease in the relative expression of these genes in the hemocytes. The relative expression levels of *Nrf1*, *Nrf2*, *GPx*, *SOD*, *CAT*, and *Hippo* were significantly elevated in the RL0.5 and RL1 groups compared to the DL2 group (*p* < 0.05). Additionally, the relative expression levels of *GPx* and *SOD* genes in the RL2 group were significantly higher than that in the DL2 group (*p* < 0.05). These findings suggest that lysophospholipids supplementation at concentrations of 0.5% to 1% can markedly enhance the relative expression levels of antioxidant genes in the hemocytes of shrimp.

### 3.3. Activity of Intestinal Digestive Enzymes

The activity of intestinal digestive enzymes in shrimp is shown in Table 4. The trypsin activity was observed to be highest in the RL1 group, with a statistically significant increase compared to other groups (*p* < 0.05). Conversely, the trypsin activity in the RL1.5 and RL2 groups was significantly lower than that in the RL0 group (*p* < 0.05). The AMS activity in the RL0.5 and RL1 groups was significantly elevated compared to the RL0 group (*p* < 0.05). Conversely, the LPS activity in the RL1.5 and RL2 groups was significantly reduced relative to the DL2 and RL0 groups (*p* < 0.05). Additionally, the LPS activity in the RL0.1 group was significantly higher than that observed in the other lysophospholipid-supplemented groups (*p* < 0.05). Supplementation with high levels of lysophospholipids (1.5–2%) resulted in a significant reduction in trypsin and LPS activities in shrimp. In contrast, lysophospholipids supplementation with low levels (0.5–1%) significantly increased the activities of trypsin and AMS. 

### 3.4. Relative Expression Levels of Intestinal Digestive Enzyme Genes

**Figure 2:**
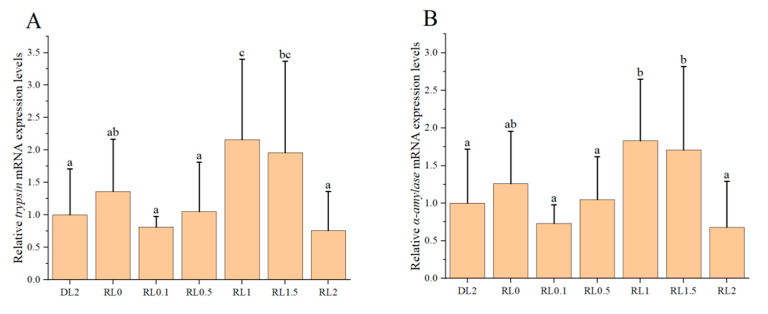
The impact of lysophospholipids on the relative expression levels of intestinal digestive enzyme genes in the shrimp. (**A**) *trypsin*; (**B**) *α-amylase*. Different lowercase letters above the bars indicate statistically significant differences (*n* = 4, *p* < 0.05).

The relative expression levels of intestinal digestive enzyme genes in the shrimp are shown in Figure 2. As the dietary lysophospholipid concentration increased from 0.1% to 2%, the relative expression of *trypsin* and *α-amylase* genes initially rose and subsequently declined. Notably, the relative expression levels of these enzymes were significantly elevated in the RL1 and RL1.5 groups compared to the DL2 group (*p* < 0.05). The inclusion of 1–1.5% lysophospholipids in the dietary markedly enhanced the relative expression levels of *trypsin* and *α-amylase* genes in the shrimp.

### 3.5. Analysis of Data by Means of Pearson Correlation

**Figure 3:**
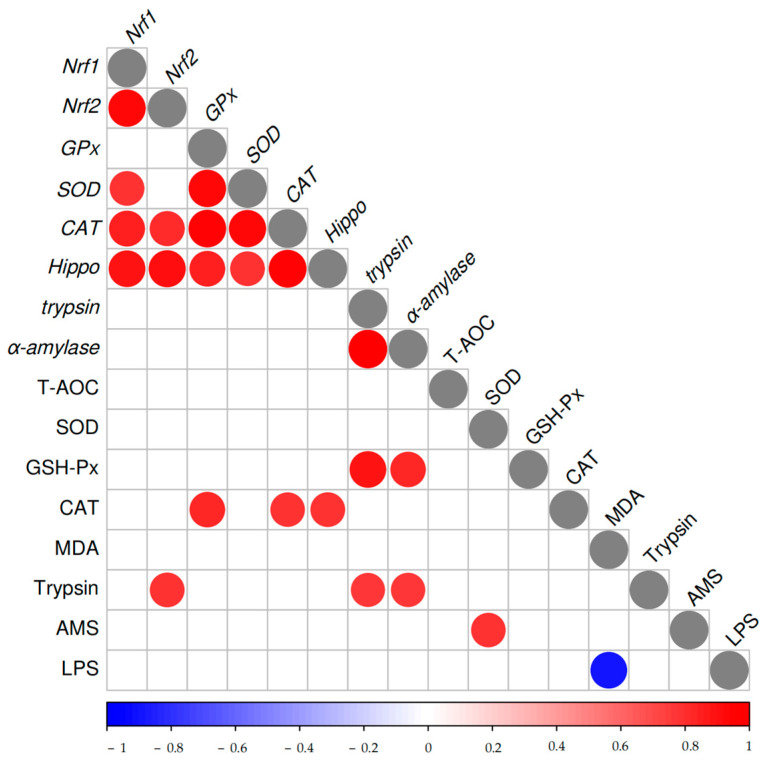
Karl–Pearson correlation plot between antioxidant and digestion-related enzyme activities and gene indicators.

A Pearson correlation analysis was performed to examine the relationships between antioxidant and digestion-related enzyme activities and gene indicators in seven different treatment groups (*n* = 4), as illustrated in Figure 3. The gene-to-gene correlation analysis revealed that *Nrf1* was positively correlated with *Nrf2*, *SOD*, *CAT*, and *Hippo*, with correlation coefficients ranging from 0.79 to 0.92. Similarly, *Nrf2* exhibited positive correlations with *CAT* and Hippo (r = 0.81~0.91), while *GPx* showed strong correlations with *SOD*, *CAT*, and *Hippo* (r = 0.84~0.94). Furthermore, *SOD* was positively correlated with *CAT* and *Hippo* (r = 0.79~0.92); *CAT* demonstrated a strong correlation with *Hippo* (r = 0.95), and *trypsin* was highly correlated with *α-amylase* (r = 0.99). In the analysis of enzyme activity-to-gene correlations, GSH-PX demonstrated a positive correlation with *trypsin* and *α-amylase*, with correlation coefficients ranging from 0.83 to 0.89. CAT was positively correlated with *GPx*, *CAT*, and *Hippo*, with correlation coefficients ranging from 0.78 to 0.83. Additionally, trypsin showed positive correlations with *Nrf2*, *trypsin*, and *α-amylase* (r = 0.76~0.79). In the enzyme activity-to-enzyme activity correlations, SOD was positively correlated with AMS (r = 0.78), whereas MDA exhibited a negative correlation with LPS (r = −0.89).

### 3.6. Intestinal Microbiota Community Changes

#### 3.6.1. Microbiota Diversity and Composition Changes

**Figure 4:**
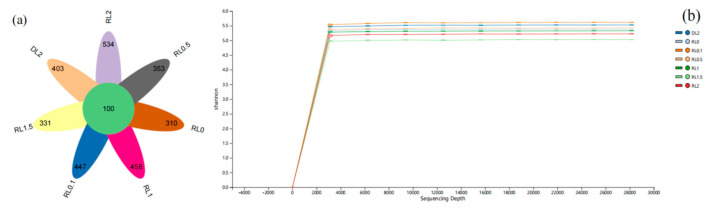
Composition and rationality analysis of ASVs. Note: In the microbial diversity assessment, after removing erroneous sequences, a 100% clustering threshold was applied, leading to the assignment of ASVs to all sequences. Typically, each ASV corresponds to a precise sequence. (**a**): Venn Diagram; (**b**): Shannon Index Curve.

The intestinal microbiome’s amplicon sequence variants (ASVs) were analyzed, as depicted in Figure 4a. A total of 1,200,196 high-quality sequences were obtained from the intestinal microbiota of *L. vannamei*. The sequence lengths varied from 230 to 437 bp, with 99.95% of the sequences ranging between 405 and 431 bp. Utilizing 16S rDNA high-throughput sequencing, a total of 4283 ASVs were identified. Of these, 100 ASVs were found to be common across all samples. Notably, the RL0 group sample exhibited the lowest number of unique ASVs, whereas the RL2 group sample demonstrated the highest number of unique ASVs.

The Shannon index curve demonstrates that with the random sampling of additional ASVs, the curve progressively levels off, suggesting that the sequencing depth within the samples is adequate (Figure 4b). These findings imply that microbial diversity is sufficiently captured.

**Figure 5:**
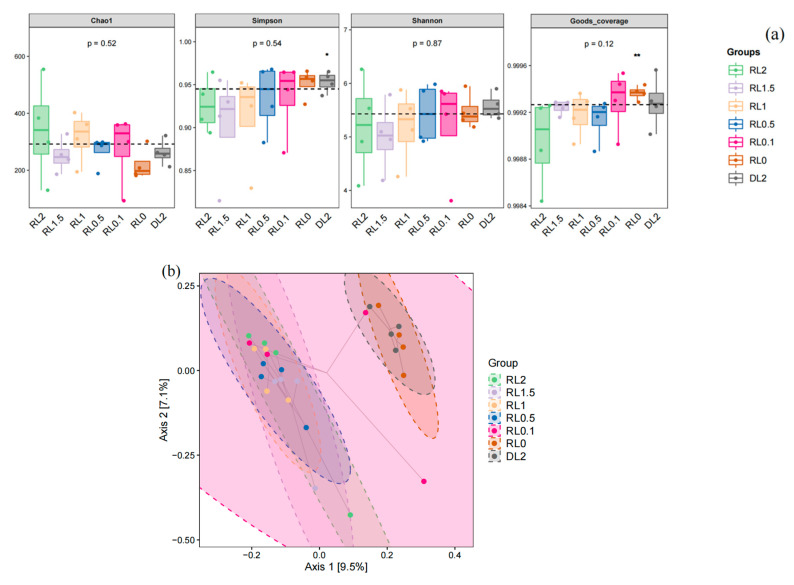

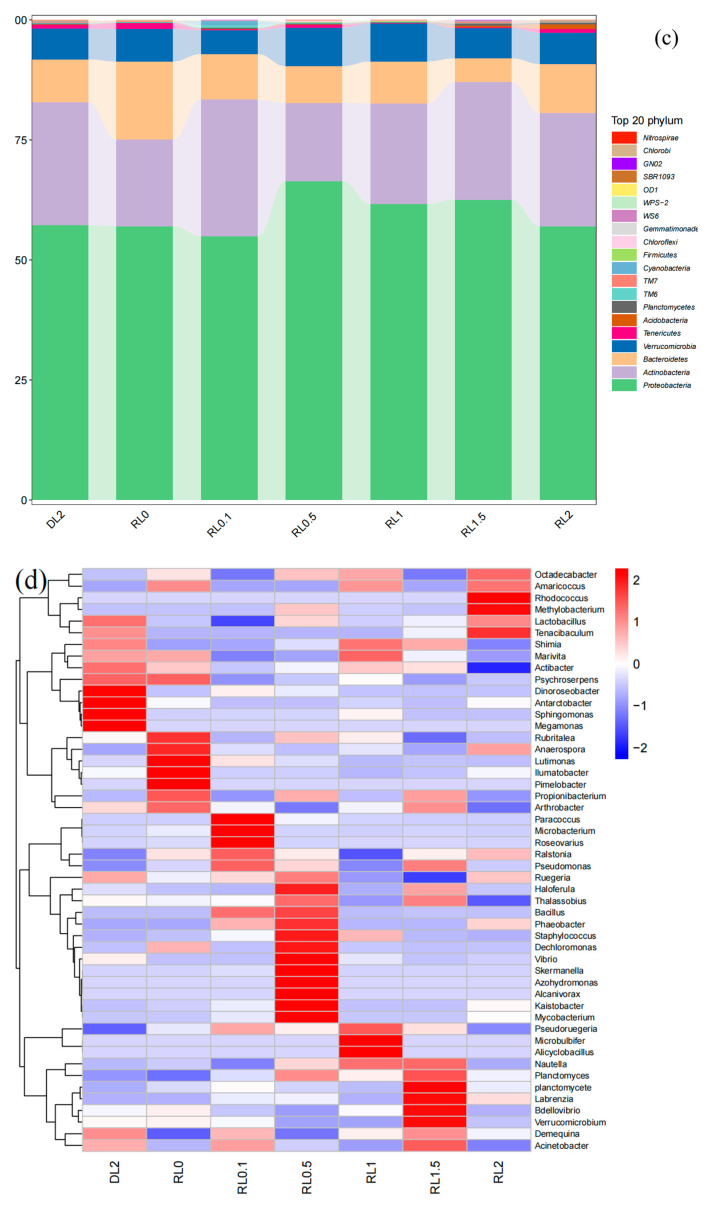
Alterations in the diversity and composition of the intestinal microbiota community in *L. vannamei* following different feed treatments. (**a**) α-diversity indices. “*, **” indicates a significant difference between this group and the other groups. (**b**) The PCoA plot depicting β-diversity. (**c**) Proportional representation of bacterial phyla abundance. (**d**) Proportional representation of bacterial genus abundance. Red colors show the increased bacterial abundance, and blue colors indicate the decreased bacterial abundance.

The microbial community coverage rate for each group surpassed 99.9%, demonstrating that the sequencing depth for the intestinal microbiome analysis was adequate. Consequently, the experimental results can be considered representative. The analysis of bacterial richness revealed an increase in the Chao1 index for the RL1 and RL2 groups relative to the control group. However, these differences were not significant (*p* > 0.05). With increasing levels of lysophospholipid supplementation, there was a general increase in the Chao1 index, whereas the Simpson and Shannon indices exhibited a general decline (Figure 5). This suggests that the addition of lysophospholipids at concentrations exceeding 1% may lead to a reduction in the intestinal microbiota diversity of *L. vannamei*. The β-diversity based on PCoA analysis, as measured by Jaccard distance, indicates that the RL0.5, RL1, RL1.5, and RL2 groups exhibit distinguishing variances of the microbial community from DL2 and RL0 groups (*p* < 0.05). In contrast, the RL0.1 group does not demonstrate significant differences when compared to the other groups.

The relative proportions of predominant bacteria underwent changes. At the phylum level, Proteobacteria and Actinobacteria constituted the core microbiota, as illustrated in Figure 5c. Proteobacteria exhibited the highest abundance in the RL0.5 group, comprising 66.29% of the microbiota, whereas Actinobacteria were most prevalent in the RL0.1 group, representing 28.59%. In contrast, Bacteroidetes were predominantly found in the RL0 group, accounting for 16.17%. Moreover, Bacteroidetes in the RL0 group were significantly higher in abundance than those in the RL1.5 group (*p* < 0.05). Notably, aside from Bacteroidetes, the top ten phyla did not demonstrate statistically significant differences across the groups (*p* > 0.05) (Table S1).

At the genus level, *Nautella*, *Demequina*, and *Ruegeria* constituted the core microbiota. *Nautella* exhibited the highest abundance in the RL1 and RL1.5 groups, representing 31.14% and 31.78% of the microbiota, respectively. *Demequina* was predominantly found in the DL2, RL0.1, and RL1.5 groups, accounting for 21.76%, 20.66%, and 21.88%, respectively. *Ruegeria* was most abundant in the RL0.5 group, comprising 18.00% of the microbiota. Notable, the RL1 and RL1.5 groups demonstrated a higher abundance of dominant species compared to the other groups. Although *Ralstonia* was most abundant in the RL0.1 group, its abundance did not differ significantly from the other groups (*p* > 0.05). *Lutimonas* exhibited the highest abundance in the RL0 group, with levels significantly surpassing those observed in the other groups (*p* < 0.05). Conversely, *Microbacterium* demonstrated the greatest abundance in the RL0.1 group, also significantly exceeding the levels found in the other groups (*p* < 0.05) (Figure 5d, Table S2).

#### 3.6.2. Variations in the Phenotypes of Intestinal Bacteria

**Figure 6:**
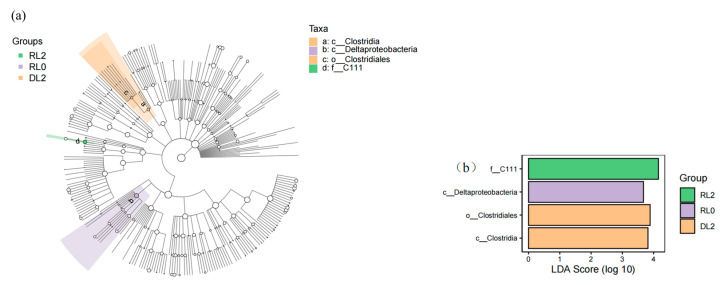
Indicator species analysis and metabolic predictions of the intestinal microbiota of *L. vannamei* following different feed treatments. (**a**) Lefse cladogram. The cladogram was obtained by mapping differences onto a known hierarchical classification tree. Orange: bacteria enriched in the DL2 group; purple: bacteria enriched in the RL0 group; green: bacteria enriched in the RL2 group; white: no significant difference. (**b**) LDA score of Lefse-PICRUSt. The length of the column represents the effect size of bacterial lineages.

The Lefse analysis employs linear discriminant analysis (LDA) effect size to assess the differential abundance of microbial taxa across groups. The cladogram illustrates that the order Clostridiales is classified under the class Clostridia, suggesting its potential as a biomarker for the DL2 group. Additionally, the family C111 is identified as a potential biomarker for the RL2 group (Figure 6a). The Lefse LDA analysis indicated that the DL2 group exhibited significant enrichment in both the class Clostridia and one order Clostridiales. In contrast, the RL0 group demonstrated enrichment in the class Deltaproteobacteria, while the RL2 group was notably enriched with the family C111 (Figure 6b).

#### 3.6.3. Prediction of the Functional Abundance of Intestinal Microbiome

**Figure 7:**
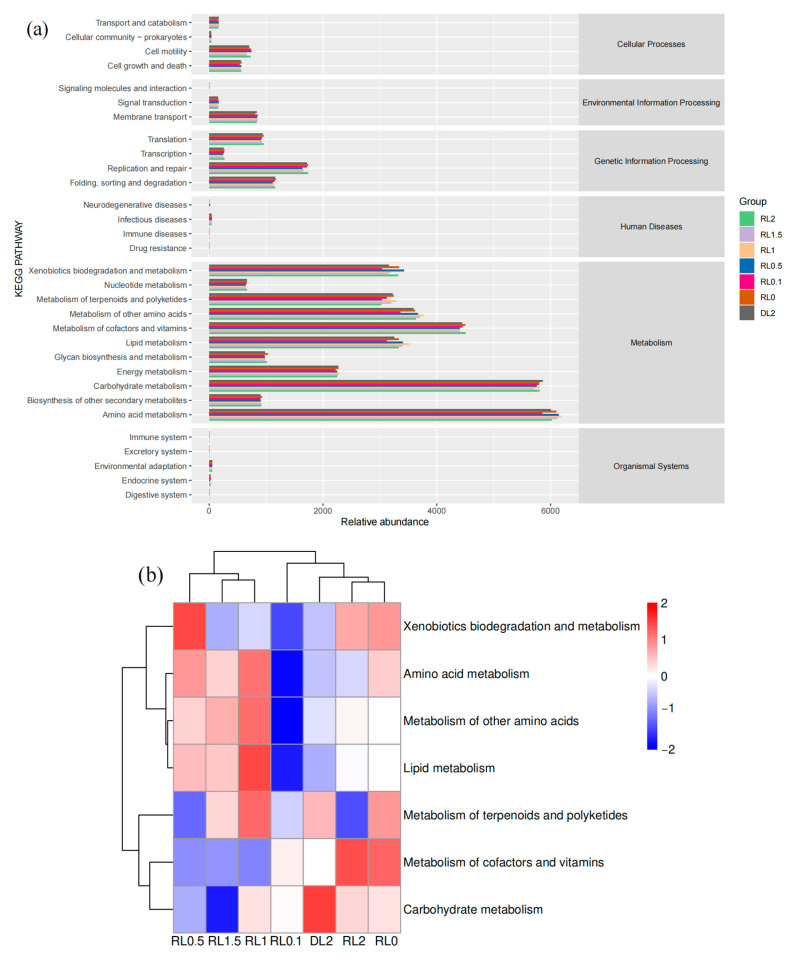
(**a**) Prediction of the functional attributes of the intestinal microbiome in *L. vannamei* using KEGG level 1 pathway. (**b**) Anticipating the primary metabolic functions of the intestinal microbiome in *L. vannamei* across varying levels of lysophospholipid supplementation as analyzed through KEGG level 2 pathways.

The functional abundance enrichment map of the intestinal microbiome (Figure 7) demonstrated a predominant enrichment in metabolic pathways, with a primary emphasis on amino acid metabolism, carbohydrate metabolism, lipid metabolism, cofactor and vitamin metabolism, terpenoid and polyketide metabolism, as well as biodegradation and metabolism of xenobiotics. Cluster heatmap analysis of the principal metabolic pathways indicated that the RL0.5, RL1, and RL1.5 groups generally displayed an elevated proportion of pathways related to amino acid metabolism, other amino acid metabolism, and secondary lipid metabolism.

#### 3.6.4. The Relationship Between Intestinal Microbiota and Host Gene Expression

**Figure 8:**
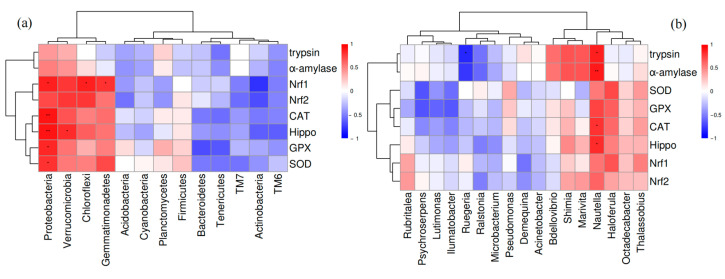
The relationship between intestinal microbiota and gene expressions in both the hemocytes and intestines of *L. vannamei* following lysophospholipid supplementation. (**a**) Correlation between intestinal microbiota phyla and gene expressions in both hemocyte and intestine. (**b**) Correlation between intestinal microbiota genera and gene expressions in both hemocyte and intestine. Positive correlations are indicated in red, whereas negative correlations are depicted in blue. Asterisks denote statistically significant differences with * *p* < 0.05 and ** *p* < 0.01.

The relationship between intestinal microbiota and gene expressions in both hemocytes and intestines was examined (Figure 8). At the phylum level, Proteobacteria demonstrated a positive correlation with the expression of antioxidant genes (*Nrf1*, *CAT*, *Hippo*, *GPX*, and *SOD*), Verrucomicrobia was positively correlated with *Hippo* expression, and both Chloroflexi and Gemmatimonadetes were positively correlated with *Nrf1* expression. At the genus level, *Nautella* exhibited a positive correlation with the expression of antioxidant genes (*CAT* and *Hippo*) as well as digestive genes (*trypsin* and *α-amylase*), whereas *Ruegeria* showed a negative correlation with *α-amylase* expression.

## 4. Discussion

This study elucidates the critical role of lysophospholipids in augmenting antioxidant capacity. Investigating the antioxidant capacity of shrimp holds substantial significance, particularly for advancing the health and productivity of shrimp aquaculture [21]. The antioxidant enzyme system serves as an essential defense mechanism against oxidative stress in shrimp, effectively neutralizing excess reactive oxygen species (ROS) through the synergistic action of multiple enzymes, including SOD, CAT, and GSH-Px, thereby safeguarding tissues from oxidative damage [22]. MDA serves as a critical biomarker for assessing lipid peroxidation, given its role as a secondary byproduct of this process that can adversely impact the structural integrity and functionality of cellular membranes. It is frequently employed to gauge the degree of oxidative damage sustained by cells and tissues [23]. Meanwhile, T-AOC is a pivotal parameter for evaluating the comprehensive efficacy of the body’s antioxidant defense mechanisms [24]. Consequently, mitigating oxidative damage and augmenting antioxidant capacity are crucial. Antioxidant enzymes present in the hemolymph are instrumental in counteracting oxidative stress and preserving the redox balance within cells [25]. In this study, the incorporation of 0.1% lysophospholipids markedly enhanced the activities of hemolymph SOD and GSH-Px, while reducing MDA levels. These results align with the findings of Weng et al. [16]. This effect may be attributed to an optimal level of lysophospholipids, which enhances fat absorption and reduces the accumulation of lipid metabolites in the hemolymph, thereby mitigating lipid peroxidation in the body [26]. As the concentration of lysophospholipids increased, T-AOC activity decreased, while GSH-Px and CAT activities initially increased and then decreased. Meanwhile, MDA activity rose, indicating a decline in antioxidant capacity. A similar outcome was observed by Xu et al. [27], who observed that the addition of 4% phospholipids to the diets of juvenile mud crabs yielded comparable results. High concentrations of emulsifiers have the potential to elevate oxidative stress and augment the production of excessive ROS. This study also identified an inverse relationship between MDA and LPS activity, indicating that when the antioxidant defense system is unable to effectively neutralize excessive ROS, there is a subsequent decline in metabolic enzyme activity. The decline can result in damage to DNA, proteins, and lipids, ultimately culminating in cell death and tissue injury [28]. Furthermore, the study revealed that the activity patterns of hemolymph SOD, CAT, and GSH-Px were consistent with the mRNA expression levels of these enzymes.

Following the incorporation of 0.5% and 1% lysophospholipids into the feed, a significant upregulation of *Nrf1*, *Nrf2*, *GPx*, *SOD*, *CAT*, *HSP70*, and *Hippo* genes was observed in the hemocytes. *Nrf1* and *Nrf2*, which are nuclear factor erythroid 2-related factors, facilitate the expression of various antioxidant enzymes, including SOD, CAT, and GPx, by binding to antioxidant response elements (AREs). Collectively, these genes contribute to antioxidant defense and cell protection [29]. Under typical physiological conditions, *Nrf1* predominantly resides within the endoplasmic reticulum. However, in response to stress, *Nrf1* is capable of translocating to the nucleus, where it activates the transcription of antioxidant genes [30]. This study identified a positive correlation between *Nrf1* and *Nrf2*, *SOD*, *CAT*, and *Hippo*. Within the Keap1-Nrf2-ARE signaling axis, Keap1 ordinarily interacts with Nrf2, facilitating its ubiquitination and degradation, thus suppressing the activity of Nrf2. Under conditions of stress, Nrf2 dissociates from the Keap1 complex, interacts with cysteine residues within the Keap1 protein, and subsequently translocates to the nucleus to initiate the expression of antioxidant genes driven by the ARE [31]. The Hippo signaling pathway is integral to the regulation of cell proliferation and apoptosis and is implicated in modulating antioxidant responses. The Hippo pathway influences the expression of antioxidant enzyme genes and enhances resistance to environmental stress by modulating downstream YAP/TAZ transcription factors [32]. In this study, a positive correlation was observed between *the* Hippo pathway and the antioxidant enzymes *GPx*, *SOD*, and *CAT*. Previous research has identified an interaction between Nrf2 and the Hippo pathway, suggesting a collaborative regulation of cellular antioxidant defense mechanisms. Specifically, Nrf2 can indirectly modulate the expression of antioxidant enzymes through the regulation of YAP/TAZ activity [33]. In the present study, Nrf2 was also found to be positively correlated with CAT and Hippo. Palliyath et al. [34] demonstrated that the Nrf1/ARE pathway is crucial in modulating the expression of antioxidant enzyme genes in shrimp. Their findings indicated that exposure to ammonia nitrogen stress markedly upregulates *Nrf1* expression, thereby augmenting antioxidant enzyme activity and enhancing the overall antioxidant capacity of shrimp. However, there is a paucity of research concerning the direct effects of emulsifiers on the Nrf1/ARE signaling pathway. Dietary additives, including yeast polysaccharide A, have the potential to enhance shrimp immunity through the modulation of TLR signaling pathways, which may, in turn, exert an indirect influence on the activity of the Nrf1/ARE pathway [35]. Furthermore, natural emulsifiers such as curcumin and polyphenolic compounds found in garlic extracts have been shown to significantly enhance antioxidant capacity by activating the Keap1-Nrf2-ARE signaling pathway [36]. This study indicates that the upregulation of antioxidant-related genes in *L. vannamei*, following the supplementation of 0.5% and 1% lysophospholipids, may contribute to the maintenance of overall health by enhancing free radical scavenging activity and providing protection against oxidative damage [37]. However, with further increases in lysophospholipid supplementation, a downregulation of antioxidant gene expression in the hemocytes was observed, paralleling results reported in rainbow trout administered a diet containing 0.3% phosphatidylcholine [17].

Lysophospholipids exert a substantial influence on the activity of digestive enzymes in aquatic animals. The present study demonstrates that relative to the control group, the activities of Trypsin and AMS were significantly enhanced with the inclusion of 0.1%, 0.5%, and 1% lysophospholipids. Existing research indicates that dietary lysophospholipids can improve growth performance, digestive enzyme activity, and overall health in aquatic animals. For instance, the supplementation of lysophospholipids in juvenile largemouth bass (*Micropterus salmoides*) has been shown to significantly increase lipase and protease activities, thereby promoting growth [38]. In a separate study, the incorporation of soybean lecithin into the diet markedly improved digestive enzyme activity in striped catfish (*Pangasianodon hypophthalmus*), with notable enhancements observed in lipase and protease activities [39]. To further elucidate the impact of lysophospholipids on shrimp digestion, we performed gene expression analysis. The relative expression levels of *trypsin* and *α-amylase* were significantly elevated following the addition of 1% and 1.5% lysophospholipids. In this experiment, the alterations in trypsin and AMS activity in shrimp hemolymph were consistent with the corresponding mRNA expression levels. The findings suggest that lysophospholipids substantially enhance the digestive efficiency of *L. vannamei*.

The intestinal microbiota encompasses the entirety of microbial communities inhabiting the digestive tract of animals. These microorganisms are integral to the health and disease states of shrimp, contributing to nutrient absorption, immune regulation, pathogen defense, and metabolic processes [40]. The analysis of the 16S rRNA gene sequences within the intestinal microbiota facilitates the examination of bacterial community structure, taxonomic identification, and functional prediction. Richness and diversity serve as comprehensive indicators, reflecting the total number, variety, and distribution of bacterial species [41]. In this experiment, no significant differences were observed in the Chao1, Shannon, and Simpson indices of the microbial communities. This suggests that the incorporation of lysophospholipids into the feed did not substantially affect the diversity or abundance of the intestinal microbiota in *L. vannamei*, regardless of variations in the concentration added.

A detailed examination of the microbial community composition at the phylum level indicated that Proteobacteria, Actinobacteria, and Bacteroidetes constituted the predominant bacterial groups within the intestinal microbiota of *L. vannamei*. At the genus level, *Nautella*, *Demequina*, and *Ruegeria* were identified as the core microbiota. Notably, *Ralstonia* and *Microbacterium* exhibited the highest abundance with the addition of 0.1% lysophospholipids, whereas *Lutimonas* was the most prevalent in conditions laking lysophospholipids. The Proteobacteria phylum, predominantly consisting of Gram-negative bacteria, is among the most prevalent in the intestinal microbiota of shrimp. Certain bacteria within this phylum possess the capability to decompose complex organic substances, thereby facilitating more efficient nutrient absorption in shrimp. Previous research has demonstrated that genera such as *Pseudomonas* and *Ralstonia* are effective in the decomposition of proteins and fats, thereby enhancing the feed utilization rate in shrimp [42]. These findings align with the results of our study, which indicate optimal growth performance with the incorporation of 0.1% lysophospholipid in the feed. *Ralstonia*, classified within the Proteobacteria phylum, functions as a probiotic with the ability to stimulate the immune system of shrimp, thereby enhancing their resistance to pathogens and significantly mitigating the risk of infections. Similarly, *Pseudomonas* is known to produce antibiotic-like compounds that effectively inhibit pathogenic bacteria within aquaculture environments. Bacteria within the Actinobacteria phylum are integral to the decomposition of organic matter and cycling of nutrients, contributing to organic waste and enhancement of water quality. Additionally, they are capable of producing growth-promoting compounds, including vitamins and amino acids, which directly facilitate the growth and development of shrimp [40]. The findings of this experiment indicated that the relative abundance of *Microbacterium*, a genus within Actinobacteria, increased following the addition of lysophospholipid. Bruno et al. [42] have demonstrated that *Microbacterium* is capable of producing natural antibiotics that effectively inhibit the growth of pathogenic bacteria, thereby reducing disease incidence in shrimp. Furthermore, Bacteroidetes bacteria have been shown to significantly enhance intestinal health, improve immune function, and increase feed utilization efficiency in shrimp aquaculture. These bacteria also inhibit the proliferation of common pathogens, such as Vibrio, through mechanisms of competitive exclusion and the secretion of antimicrobial substances, consequently decreasing disease in shrimp. Under specific conditions, *Lutimonas* bacteria may transition into potential pathogens, which can adversely affect the diversity and functionality of the diversity and functionality of intestinal microbiota by excluding certain beneficial probiotics [43]. The experiment results indicated that the addition of lysophospholipid could effectively modulate *Lutimonas* levels, potentially exerting positive effects on the balance of intestinal microbiota. This study highlighted that Proteobacteria, Actinobacteria, and Bacteroidetes are the predominant bacterial groups within the shrimp intestinal microbiota, playing crucial roles in nutrient absorption, immune regulation, and pathogen inhibition in shrimp. The augmented relative abundance of *Ralstonia*, *Microbacterium*, and *Pseudomonas* following the addition of lysophospholipid suggests that lysophospholipid supplementation facilitates nutrient absorption and metabolism, enhances immune function, suppresses pathogenic bacteria, and contributes to the maintenance of intestinal health in shrimp.

Gut microbiota has the capacity to impact the host’s metabolic homeostasis through the gut–liver axis [44]. This study identified several bacterial species that exhibit significant associations with alterations in host metabolism. Specifically, the intestinal microbiota can metabolize a range of compounds, which are subsequently absorbed by the hepatopancreas, thereby influencing its metabolic functions. At the phylum level, Proteobacteria, Verrucomicrobia, Chloroflexi, and Gemmatimonadetes exhibited a positive correlation with the expression of antioxidant genes. At the genus levels, *Nautella* demonstrated a positive correlation with the expression of both antioxidant and digestive genes. These observations imply a potential association of these bacteria taxa with oxidative stress in shrimp. The observed trends suggest that supplementation with lysophospholipids may affect the equilibrium of these metabolites and is potentially associated with the metabolic processes of the shrimp hepatopancreas. Naguib et al. [45] explored oxidative stress and antioxidant defense mechanisms in Proteobacteria, highlighting the functions of antioxidant molecules such as SOD, CAT, and GPx. Certain bacteria within the phylum Verrucomicrobia exhibit resistance to oxidative stress through the synthesis of antioxidant enzymes, including SOD, CAT, and GPx. Similarly, *Nautella*, a marine bacterium classified under Alphaproteobacteria, is hypothesized to utilize analogous antioxidant mechanisms to safeguard against oxidative damage [46]. These antioxidant strategies not only provide protection from oxidative harm but may also play a crucial role in preserving the stability and efficiency of digestive processes. This study, however, identified only correlations between intestinal microbiota and host metabolism, underscoring the need for further research into the intestine-liver axis of intestinal microbiota. The significantly altered genes identified herein offer a basis for further exploration of the mechanisms underlying lysophospholipids supplementation in shrimp. In relation to intestinal bacteria, these findings contribute valuable insights for the development of innovative feed additives.

The functional prediction of the intestinal microbiota in *L. vannamei* indicated a predominant enrichment in metabolic pathways. Supplementation with lysophospholipids generally elevated the proportion of secondary pathways associated with amino acid and lipid metabolism. This increased proportion of metabolic pathways suggests an enhanced capacity of the shrimp intestinal microbiota to utilize feed nutrients more effectively.

## 5. Conclusions

This study illustrated that the strategic supplementation of lysophospholipids in the diet exerts a beneficial influence on the antioxidant capacity, digestive processes, and microbial composition of *L. vannamei*. The inclusion of lysophospholipids was found to enhance the activity of hemolymph antioxidant enzymes. Notable, a 0.1% supplementation of lysophospholipids significantly increased T-AOC activity in shrimp hemolymph, while concentrations ranging from 0.5% to 1.5% significantly elevated the activities of GSH-Px and CAT, as well as MDA levels. The supplementation of lysophospholipids at concentrations ranging from 0.5% to 1% resulted in a significant upregulation of antioxidant gene expression in shrimp hemocytes. Additionally, lysophospholipids at these low levels (0.5% to 1%) markedly enhanced the activities of Trypsin and AMS. Furthermore, dietary inclusion of lysophospholipids at levels between 1% and 1.5% significantly elevated the expression levels of *trypsin* and *α-amylase* genes. The supplementation of lysophospholipids also contributed to improvements in intestinal microbiota by facilitating nutrient absorption and metabolism, enhancing immune function, and maintaining intestinal health. Based on the findings of this study, the optimal dietary supplementation level of lysophospholipids for *L. vannamei* is approximately 0.1%.

## Figures and Tables

**Table 1:** Ingredients and proximate composition of the different experimental diets.

Items	DL2	RL0	RL0.1	RL0.5	RL1	RL1.5	RL2
Ingredients ^a^							
Fish meal	25.00	25.00	25.00	25.00	25.00	25.00	25.00
Soybean meal	18.00	18.00	18.00	18.00	18.00	18.00	18.00
Wheat flour	21.99	23.99	23.89	23.49	22.99	22.49	21.99
Krill meal	5.00	5.00	5.00	5.00	5.00	5.00	5.00
Peanut meal	16.40	16.40	16.40	16.40	16.40	16.40	16.40
Brewer’s yeast	5.00	5.00	5.00	5.00	5.00	5.00	5.00
Fish oil	1.00	1.00	1.00	1.00	1.00	1.00	1.00
Soybean oil	1.00	1.00	1.00	1.00	1.00	1.00	1.00
Multi-minerals ^b^	1.00	1.00	1.00	1.00	1.00	1.00	1.00
Multi-vitamins ^c^	1.00	1.00	1.00	1.00	1.00	1.00	1.00
Dicalcium phosphate	1.00	1.00	1.00	1.00	1.00	1.00	1.00
Choline chloride	0.50	0.50	0.50	0.50	0.50	0.50	0.50
Soybean phospholipids	2.00						
Lysophospholipids ^d^		0	0.1	0.5	1	1.5	2
Vc phosphate	0.10	0.10	0.10	0.10	0.10	0.10	0.10
Sodium alginate	1.00	1.00	1.00	1.00	1.00	1.00	1.00
Y_2_O_3_	0.01	0.01	0.01	0.01	0.01	0.01	0.01
Total	100.00	100.00	100.00	100.00	100.00	100.00	100.00
Nutritional Levels ^e^							
Moisture	5.10	5.40	5.80	7.80	7.80	8.93	8.70
Crude fat	6.01	5.61	5.91	6.5	6.99	7.27	7.46
Crude protein	41.28	41.34	40.63	40.23	40.14	39.04	38.38
Crude ash	10.60	10.56	10.49	10.23	10.5	10.15	10.04

Note: ^a^. Fish meal, soybean meal, wheat flour, krill meal, peanut meal, brewer’s yeast, fish oil, dicalcium phosphate, choline chloride, vc phosphate, and sodium alginate purchased from Qingdao Baiwei Yingge Biotechnology Co., Ltd. (Qingdao, China). ^b^. Purchased from Guangzhou Bauxite Aquatic Technology Co., Ltd. (Guangzhou, China). Vitamin premix (g kg^−1^): VE, 75; VK, 2.5; VB_1_, 0.25; VB_2_, 1.0; VB_3_, 5.0; VB_6_, 0.75; VB_12_, 2.5; VA, 2.5; VD, 6.25; folic acid, 0.25; cellulose, 500; inositol, 379; biotin, 2.5. ^c^. Purchased from Guangzhou Xinghailisheng Biotechnology Co., Ltd. (Guangzhou, China). Mineral premix (g kg^−1^): potassium chloride, 90; sodium chloride, 40; KI, 0.04; ZnSO_4_·7H_2_O, 4; CuSO_4_·5H_2_O, 3; CoSO_4_·7H_2_O, 0.02; MnSO_4_·H2O, 3; FeSO_4_·7H_2_O, 20; MgSO_4_·_7_H2O, 124; CaCO_3_, 215; Ca(H_2_PO_4_)_2_·2H_2_O, 500. ^d^. The lysophospholipids were obtained from Kemin Industries (Zhuhai, China). ^e^. Nutrient levels in dry feed weight.

**Table 2:** The specific primers for real-time fluorescence quantification PCR.

cDNA	Forward Primer (5′~3′)	Reverse Primer (5′~3′)	Size of Production (bp)	GenBank Nos.
*β-actin*	CGAGGTATCCTCACCCTGAA	GTCATCTTCTCGCGGTTAGC	176	AF300705.2
Antioxidant-Related Genes				
*Nrf1*	TCCCAAGAGTGAGACAAAGATT	CACCAGTCTCAGAGTCGATA	86	XM_027363478.1
*Nrf2*	TCTTGTTGGTCCCTCGCTCCTC	TCACTGCTTGGGGTCATCCTTCC	89	XM_027367070.1
*G* *P* *x*	GGCACCAGGAGAACACTAC	CGACTTTGCCGAACATAAC	102	AY973252.2
*SOD*	GCAATGAATGCCCTTCTACC	CAGAGCCTTTCACTCCAACG	199	XM_027376216.1
*CAT*	TACTGCAAGTTCCATTACAAGACG	GTAATTCTTTGGATTGCGGTCA	285	XM_027383088.1
*Hippo*	TGAGCACAACCAAACCCACCATC	CATCGTCCGACTGTCCACTTCATC	86	MW415984.1
Digestion-Related Genes				
*trypsin*	CGGAGAGCTGCCTTACCAG	TCGGGGTTGTTCATGTCCTC	141	PP817223.1
*α-amylase*	CTCTGGTAGTGCTGTTGGCT	TGTCTTACGTGGGACTGGAAG	116	AJ133526.3

Note: *Nrf1*, nuclear respiratory factor 1; *Nrf2*, nuclear factor erythroid 2-related factor 2; *GPx*, glutathione; *SOD*, superoxide dismutase; *CAT*, hydrogen peroxide.

**Table 3:** Antioxidant enzyme activities in the hemolymph of shrimp fed experimental diets containing graded levels of lysophospholipids (*n* = 4).

Items	DL2	RL0	RL0.1	RL0.5	RL1	RL1.5	RL2
T-AOC (U/mL)	2.18 ± 0.30 ^a^	2.91 ± 0.27 ^ab^	4.46 ± 0.87 ^b^	3.39 ± 0.35 ^ab^	3.42 ± 1.59 ^ab^	3.77 ± 1.00 ^ab^	2.19 ± 0.11 ^a^
SOD (U/mL)	62.05 ± 8.52 ^a^	73.61 ± 2.66 ^b^	75.61 ± 6.61 ^b^	75.25 ± 3.83 ^b^	73.07 ± 0.47 ^b^	78.84 ± 6.72 ^b^	80.69 ± 5.68 ^b^
GSH-Px (U/mL)	144.62 ± 39.80 ^a^	174.07 ± 34.89 ^a^	189.44 ± 20.10 ^a^	172.81 ± 29.56 ^a^	298.52 ± 33.04 ^b^	276.56 ± 30.84 ^b^	186.16 ± 27.88 ^a^
CAT (U/mL)	8.33 ± 0.31 ^d^	5.62 ± 0.48 ^a^	6.74 ± 0.31 ^b^	9.99 ± 0.33 ^e^	9.56 ± 0.33 ^e^	9.69 ± 0.32 ^e^	7.39 ± 0.27 ^c^
MDA (nmol/mL)	18.65 ± 1.85 ^a^	16.55 ± 1.65 ^a^	15.64 ± 2.24 ^a^	28.42 ± 2.34 ^bc^	21.69 ± 5.90 ^ab^	27.55 ± 2.01 ^bc^	30.21 ± 7.49 ^c^

Note: Different lowercase letters in the lines show significantly different data among treatments (*p* < 0.05).

**Table 4:** Intestinal digestive enzyme activities in the hemolymph of shrimp fed experimental diets containing graded levels of lysophospholipids (U/mg prot, *n* = 4).

Items	DL2	RL0	RL0.1	RL0.5	RL1	RL1.5	RL2
Trypsin	66.63 ± 3.92 ^b^	91.79 ± 8.60 ^d^	81.75 ± 11.16 ^cd^	80.02 ± 1.95 ^cd^	123.35 ± 5.48 ^e^	70.48 ± 3.63 ^bc^	48.38 ± 7.62 ^a^
AMS	1.17 ± 0.26 ^a^	2.13 ± 0.04 ^bc^	1.90 ± 0.09 ^b^	2.61 ± 0.24 ^d^	2.58 ± 0.22 ^d^	2.24 ± 0.06 ^bcd^	2.50 ± 0.17 ^cd^
LPS	3.75 ± 0.52 ^cd^	3.62 ± 0.27 ^cd^	3.98 ± 0.21 ^d^	3.37 ± 0.18 ^abc^	3.45 ± 0.16 ^bc^	2.94 ± 0.20 ^a^	3.06 ± 0.10 ^ab^

Note: Different lowercase letters in the lines show significantly different data among treatments (*p* < 0.05).

## Data Availability

Data are contained within the article.

## Supplementary Materials

The following supporting information can be downloaded at https://www.mdpi.com/article/10.3390/biology14010090/s1, Table S1: Distribution of the top 10 microbial phyla in the intestine contents of *L. vannamei* in different treatment groups; Table S2: Distribution of the top 10 microbial genera in the intestine contents of *L. vannamei* in different treatment groups.

## References

[1] Chen W., Gao S. (2023). Current Status of Industrialized Aquaculture in China: A Review. Environ. Sci. Pollut. Res..

[2] N’Souvi K., Sun C., Che B., Vodounon A. (2024). Shrimp Industry in China: Overview of the Trends in the Production, Imports and Exports during the Last Two Decades, Challenges, and Outlook. Front. Sustain. Food Syst..

[3] Liao I.C., Chien Y.-H., Galil B.S., Clark P.F., Carlton J.T. (2011). The Pacific White Shrimp, *Litopenaeus vannamei*, in Asia: The World’s Most Widely Cultured Alien Crustacean. In the Wrong Place—Alien Marine Crustaceans: Distribution, Biology and Impacts.

[4] Zhang C., Guo C.-Y., Shu K.-H., Xu S.-L., Wang D.-L. (2024). Comparative Analysis of the Growth Performance, Vitality, Body Chemical Composition and Economic Efficiency of the Main Cultivated Strains of Pacific White Shrimp (*Litopenaeus vannamei*) in Coastal Areas of China. Aquaculture.

[5] Nakano K., Ashida K. (1975). Role of Some Hormones in Protein Sparing Action of Dietary Fat. Agric. Biol. Chem..

[6] Sankian Z., Khosravi S., Kim Y.-O., Lee S.-M. (2017). Effect of Dietary Protein and Lipid Level on Growth, Feed Utilization, and Muscle Composition in Golden Mandarin Fish Siniperca Scherzeri. Fish. Aquat. Sci..

[7] Ma J., Kong L., Zhou S., Lin H., Lin Y., Qin H., Long Z., Liu L., Huang Z., Li Z. (2023). Effect of Supplementation of Chlorogenic Acid to High-Fat Diet on Growth, Lipid Metabolism, Intestinal and Hepatic Histology, and Gut Microbiota of Spotted Sea Bass (*Lateolabrax maculatus*). Metabolites.

[8] Gong H., Lawrence A.L., Jiang D.-H., Gatlin D.M. (2000). Lipid Nutrition of Juvenile *Litopenaeus vannamei*. Aquaculture.

[9] Hao Y., Guo M., Feng Y., Dong Q., Cui M. (2020). Lysophospholipids and Their G-Coupled Protein Signaling in Alzheimer’s Disease: From Physiological Performance to Pathological Impairment. Front. Mol. Neurosci..

[10] Heringdorf D.M., Offermanns S., Rosenthal W. (2008). zu Lysophospholipids. Encyclopedia of Molecular Pharmacology.

[11] Sedghi M., Javanmard F., Amoozmehr A., Zamany S., Mohammadi I., Kim W., Choppa V.S.R. (2024). Lysophospholipid Supplementation in Broiler Breeders’ Diet Benefits Offspring’s Productive Performance, Blood Parameters, and Hepatic β-Oxidation Genes. Animals.

[12] Zhang M., Bai H., Zhao Y., Wang R., Li G., Zhang G., Zhang Y. (2022). Effects of Dietary Lysophospholipid Inclusion on the Growth Performance, Nutrient Digestibility, Nitrogen Utilization, and Blood Metabolites of Finishing Beef Cattle. Antioxidants.

[13] Grove S.S., Dall J., Madsen J.G. (2024). The Effect of Lysophospholipids and Sex on Growth Performance and Small Intestine Morphology in Weanling Pigs, 7–30 Kg. Animals.

[14] Li B., Li Z., Sun Y., Wang S., Huang B., Wang J. (2019). Effects of Dietary Lysolecithin (LPC) on Growth, Apparent Digestibility of Nutrient and Lipid Metabolism in Juvenile Turbot Scophthalmus *Maximus * L.. Aquac. Fish..

[15] Liu G., Ma S., Chen F., Gao W., Zhang W., Mai K. (2020). Effects of Dietary Lysolecithin on Growth Performance, Feed Utilization, Intestinal Morphology and Metabolic Responses of Channel Catfish (*Ictalurus punctatus*). Aquacult Nutr..

[16] Weng M., Zhang W., Zhang Z., Tang Y., Lai W., Dan Z., Liu Y., Zheng J., Gao S., Mai K. (2022). Effects of Dietary Lysolecithin on Growth Performance, Serum Biochemical Indexes, Antioxidant Capacity, Lipid Metabolism and Inflammation-Related Genes Expression of Juvenile Large Yellow Croaker (*Larimichthys crocea*). Fish Shellfish. Immunol..

[17] Taghavizadeh M., Hosseini Shekarabi S.P., Mehrgan M.S., Islami H.R. (2020). Efficacy of Dietary Lysophospholipids (Lipidol^TM^) on Growth Performance, Serum Immuno-Biochemical Parameters, and the Expression of Immune and Antioxidant-Related Genes in Rainbow Trout (*Oncorhynchus mykiss*). Aquaculture.

[18] Yan M., Wang W., Huang X., Wang X., Wang Y. (2020). Interactive Effects of Dietary Cholesterol and Phospholipids on the Growth Performance, Expression of Immune-Related Genes and Resistance against Vibrio Alginolyticus in White Shrimp (*Litopenaeus vannamei*). Fish Shellfish. Immunol..

[19] Ming J., Ye J., Zhang Y., Yang X., Shao X., Qiang J., Xu P. (2019). Dietary Optimal Reduced Glutathione Improves Innate Immunity, Oxidative Stress Resistance and Detoxification Function of Grass Carp (*Ctenopharyngodon idella*) against Microcystin-LR. Aquaculture.

[20] Livak K.J., Schmittgen T.D. (2001). Analysis of Relative Gene Expression Data Using Real-Time Quantitative PCR and the 2−LiangMethod. Methods.

[21] Zhang L., Zhang P., Tan P., Xu D., Wang L., Ding Z., Shao Q. (2024). Yarrowia Lipolytica as a Promising Protein Source for Pacific White Shrimp (*Litopenaeus vannamei*) Diet: Impact on Growth Performance, Metabolism, Antioxidant Capacity, and Apparent Digestibility. Front. Mar. Sci..

[22] Qiao X.-L., Liang Q.-J., Liu Y., Wang W.-N. (2020). A Novel Kelch-Like-1 Is Involved in Antioxidant Response by Regulating Antioxidant Enzyme System in Penaeus Vannamei. Genes.

[23] Ayala A., Muñoz M.F., Argüelles S. (2014). Lipid Peroxidation: Production, Metabolism, and Signaling Mechanisms of Malondialdehyde and 4-Hydroxy-2-Nonenal. Oxidative Med. Cell. Longev..

[24] Ye Y., Zhu B., Yun J., Yang Y., Tian J., Xu W., Du X., Zhao Y., Li Y. (2024). Comparison of Antioxidant Capacity and Immune Response between Low Salinity Tolerant Hybrid and Normal Variety of Pacific White Shrimp (*Litopenaeus vannamei*). Aquacult Int..

[25] Zhu J., Shi W., Zhao R., Gu C., Li H., Wang L., Wan X. (2024). Effects of Cold Stress on the Hemolymph of the Pacific White Shrimp Penaeus Vannamei. Fishes.

[26] Holme M.-H., Southgate P.C., Zeng C. (2007). Assessment of Dietary Lecithin and Cholesterol Requirements of Mud Crab, Scylla Serrata, Megalopa Using Semi-Purified Microbound Diets. Aquac. Nutr..

[27] Xu H., Liu T., Feng W., He J., Han T., Wang J., Wang C. (2023). Dietary Phosphatidylcholine Improved the Survival, Growth Performance, Antioxidant, and Osmoregulation Ability of Early Juvenile Mud Crab Scylla Paramamosain. Aquaculture.

[28] Ponnampalam E.N., Kiani A., Santhiravel S., Holman B.W.B., Lauridsen C., Dunshea F.R. (2022). The Importance of Dietary Antioxidants on Oxidative Stress, Meat and Milk Production, and Their Preservative Aspects in Farm Animals: Antioxidant Action, Animal Health, and Product Quality—Invited Review. Animals.

[29] Chen F., Xiao M., Hu S., Wang M. (2024). Keap1-Nrf2 Pathway: A Key Mechanism in the Occurrence and Development of Cancer. Front. Oncol..

[30] Wang Z., Aweya J.J., Yao D., Zheng Z., Wang C., Zhao Y., Li S., Zhang Y. (2022). Taurine Metabolism Is Modulated in Vibrio-Infected Penaeus Vannamei to Shape Shrimp Antibacterial Response and Survival. Microbiome.

[31] Tian W., Rojo De La Vega M., Schmidlin C.J., Ooi A., Zhang D.D. (2018). Kelch-like ECH-Associated Protein 1 (KEAP1) Differentially Regulates Nuclear Factor Erythroid-2–Related Factors 1 and 2 (NRF1 and NRF2). J. Biol. Chem..

[32] Ibrahim L., Mesgarzadeh J., Xu I., Powers E.T., Wiseman R.L., Bollong M.J. (2020). Defining the Functional Targets of Cap‘n’Collar Transcription Factors NRF1, NRF2, and NRF3. Antioxidants.

[33] Sykiotis G.P. (2021). Keap1/Nrf2 Signaling Pathway. Antioxidants.

[34] Palliyath G.K., Jangam A.K., Katneni V.K., Kaikkolante N., Panjan Nathamuni S., Jayaraman R., Jagabattula S., Moturi M., Shekhar M.S. (2024). Meta-Analysis to Unravel Core Transcriptomic Responses in Penaeus Vannamei Exposed to Biotic and Abiotic Stresses. Biochem. Genet..

[35] Sangklai N., Supungul P., Jaroenlak P., Tassanakajon A. (2024). Immune Signaling of *Litopenaeus vannamei* C-Type Lysozyme and Its Role during Microsporidian Enterocytozoon Hepatopenaei (EHP) Infection. PLoS Pathog..

[36] Serafini M.M., Catanzaro M., Fagiani F., Simoni E., Caporaso R., Dacrema M., Romanoni I., Govoni S., Racchi M., Daglia M. (2020). Modulation of Keap1/Nrf2/ARE Signaling Pathway by Curcuma- and Garlic-Derived Hybrids. Front. Pharmacol..

[37] Li S., Li H., Xu X., Saw P.E., Zhang L. (2020). Nanocarrier-Mediated Antioxidant Delivery for Liver Diseases. Theranostics.

[38] Wang S., Han Z., Turchini G.M., Wang X., Fang Z., Chen N., Xie R., Zhang H., Li S. (2022). Effects of Dietary Phospholipids on Growth Performance, Digestive Enzymes Activity and Intestinal Health of Largemouth Bass (*Micropterus salmoides*) Larvae. Front. Immunol..

[39] Amer A.-R., Eweedah N.M., Amer A.A., Gewaily M.S., Younis N.A., Ahmed H.A., Dawood M.A.O. (2023). Dietary Effect of Soybean Lecithin on the Growth Performance, Digestive Enzyme Activity, Blood Biomarkers, and Antioxidative Status of Striped Catfish, *Pangasianodon hypophthalmus*. PLoS ONE.

[40] El-Saadony M.T., Shehata A.M., Alagawany M., Abdel-Moneim A.-M.E., Selim D.A., Abdo M., Khafaga A.F., El-Tarabily K.A., El-Shall N.A., Abd El-Hack M.E. (2022). A Review of Shrimp Aquaculture and Factors Affecting the Gut Microbiome. Aquacult Int..

[41] Egerton S., Culloty S., Whooley J., Stanton C., Ross R.P. (2018). The Gut Microbiota of Marine Fish. Front. Microbiol..

[42] Bruno A., Sandionigi A., Panio A., Rimoldi S., Orizio F., Agostinetto G., Hasan I., Gasco L., Terova G., Labra M. (2023). Aquaculture Ecosystem Microbiome at the Water-Fish Interface: The Case-Study of Rainbow Trout Fed with Tenebrio Molitor Novel Diets. BMC Microbiol..

[43] Duan Y., Wang Y., Ding X., Xiong D., Zhang J. (2020). Response of Intestine Microbiota, Digestion, and Immunity in Pacific White Shrimp *Litopenaeus vannamei* to Dietary Succinate. Aquaculture.

[44] Hu C., Liu M., Wan T., Tang L., Sun B., Zhou B., Lam J.C.W., Lam P.K.S., Chen L. (2021). Disturbances in Microbial and Metabolic Communication across the Gut–Liver Axis Induced by a Dioxin-like Pollutant: An Integrated Metagenomics and Metabolomics Analysis. Environ. Sci. Technol..

[45] Naguib M., Feldman N., Zarodkiewicz P., Shropshire H., Biamis C., El-Halfawy O.M., McCain J., Dezanet C., Décout J.-L., Chen Y. (2022). An Evolutionary Conserved Detoxification System for Membrane Lipid-Derived Peroxyl Radicals in Gram-Negative Bacteria. PLoS Biol..

[46] DeLong E.F. (2005). Microbial Community Genomics in the Ocean. Nat. Rev. Microbiol..

