# Supplementary material for: Effects of Lysophospholipids on the Antioxidant Capacity, Digestive Performance, and Intestinal Microbiota of Litopenaeus vannamei
Source: Biology (Basel). 2025 Jan 17;14(1):90. doi: 10.3390/biology14010090 (PMC11761955; doi:10.3390/biology14010090)
Supplement: Supplementary file 1 [file biology-14-00090-s001.zip › biology-3405399-supplementary.pdf]

Table S1. Distribution of the top 10 microbial phyla in the intestine contents of *L. vannamei* in different treatment groups (n = 4).

Phylum	DL2	RL0	RL0.1	RL0.5	RL1	RL1.5	RL2
Proteobacteria	57.22±13.55	57.05±9.25	54.85±8.40	66.29±12.72	61.71±15.48	62.57±8.67	56.89±16.16
Actinobacteria	25.51±11.02	18.18±9.02	28.59±10.84	16.35±10.13	20.97±9.98	24.64±3.39	23.66±15.49
Bacteroidetes	8.96±5.07^{ab}	16.17±3.63^{b}	9.33±2.56^{ab}	7.61±3.19^{ab}	8.73±5.15^{ab}	4.83±2.88^{a}	10.18±10.08^{ab}
Verrucomicrobia	6.45±1.97	6.66±2.43	5.02±4.67	8.11±2.86	7.86±4.74	6.25±3.69	6.60±2.45
Tenericutes	0.84±1.10	1.25±1.38	0.17±0.19	0.58±0.54	0.02±0.02	0.18±0.19	0.76±1.39
Acidobacteria	0.03±0.03	0.02±0.02	0.05±0.04	0.13±0.22	0.02±0.01	0.36±0.71	0.99±1.88
Planctomycetes	0.18±0.12	0.11±0.05	0.27±0.30	0.15±0.05	0.20±0.17	0.39±0.36	0.27±0.27
TM6	0.19±0.21	0.13±0.25	0.61±1.06	0.06±0.07	0.02±0.03	0.12±0.21	0.13±0.15
TM7	0.31±0.50	0.21±0.31	0.03±0.03	0.03±0.05	0.06±0.07	0.37±0.50	0.23±0.40
Cyanobacteria	0.01±0.01	0.00±0.00	0.80±1.56	0.10±0.18	0.02±0.01	0.01±0.01	0.04±0.05

Note: Different lowercase letters in the lines show significantly different data among treatments ($p < 0.05$).

Table S2. Distribution of the top 10 microbial genera in the intestine contents of *L. vannamei* in different treatment groups (n = 4).

Genus	DL2	RL0	RL0.1	RL0.5	RL1	RL1.5	RL2
Nautella	17.19±9.45	18.35±10.45	13.37±2.48	24.48±14.39	31.14±19.97	31.78±14.14	16.11±11.76
Demequina	21.76±10.80	12.81±8.60	20.66±9.55	13.44±8.83	18.58±10.04	21.88±3.67	17.77±11.75

Ruegeria	16.88±3.11	14.54±2.25	15.70±2.50	18.00±2.45	12.52±5.56	10.41±4.89	16.21±11.34
Ralstonia	2.60±2.86	6.04±5.24	8.98±15.95	5.80±5.39	1.58±1.52	5.87±5.53	6.93±13.14
Octadecabacter	2.74±1.43	4.41±3.03	1.53±1.45	5.05±0.78	5.55±7.80	1.44±1.36	6.67±4.75
Haloferula	2.84±0.65	2.65±0.78	2.65±1.75	4.22±3.96	2.56±1.82	3.48±2.05	2.74±1.33
Lutimonas	1.63±0.79[a]	9.17±5.45[b]	3.39±4.40[a]	1.76±1.05[a]	0.83±0.36[a]	1.14±0.93[a]	1.00±0.69[a]
Thalassobius	1.49±1.22	1.44±0.40	1.48±0.65	2.23±0.95	0.97±0.72	2.08±3.54	0.69±0.81
Rubritalea	0.73±0.22	1.43±1.55	0.49±0.51	0.97±0.30	0.78±0.95	0.18±0.13	0.51±0.57
Microbacterium	0.09±0.09[a]	0.33±0.23[a]	3.50±5.04[b]	0.07±0.07[a]	0.04±0.06[a]	0.09±0.11[a]	0.03±0.03[a]

Note: Different lowercase letters in the lines show significantly different data among treatments ($p < 0.05$).
